# Clinical impact of ulceration width, lymphovascular invasion, microscopic satellitosis, perineural invasion, and mitotic rate in patients undergoing sentinel lymph node biopsy for cutaneous melanoma: a retrospective observational study at a comprehensive cancer center

**DOI:** 10.1002/cam4.1320

**Published:** 2018-02-21

**Authors:** Kenjiro Namikawa, Phyu P. Aung, Jeffrey E. Gershenwald, Denái R. Milton, Victor G. Prieto

**Affiliations:** ^1^ Department of Pathology The University of Texas MD Anderson Cancer Center Houston Texas; ^2^ Department of Dermatologic Oncology National Cancer Center Hospital Tokyo Japan; ^3^ Department of Surgical Oncology The University of Texas MD Anderson Cancer Center Houston Texas; ^4^ Department of Biostatistics The University of Texas MD Anderson Cancer Center Houston Texas

**Keywords:** Lymphovascular invasion, melanoma, microscopic satellitosis, mitotic rate, prognosis, ulceration width

## Abstract

The prognostic significance of the width of the ulceration in primary melanomas remains unclear, and there is a relative paucity of data for lymphovascular invasion (LVI), microscopic satellitosis (MS), perineural invasion (PNI), and mitotic rate when compared with other pathological elements currently required for reporting. To evaluate the prognostic importance of the ulceration width and other important pathologic measurements, a single‐institutional retrospective study was conducted using records of cutaneous melanoma patients who underwent sentinel lymph node (SLN) biopsy at The University of Texas, MD Anderson Cancer Center between 2003 and 2008. We identified 1898 eligible patients with median tumor thickness of 1.25 mm and median follow‐up of 6.7 years. By multivariable analyses, the strongest risk factor for SLN positivity was high tumor thickness followed by the presence of LVI. The pathologic measures with the strongest influence on recurrence‐free survival (RFS) were tumor thickness and positive SLN status. Ulceration width and presence of MS were also significantly associated with RFS while PNI was not. Factors with the strongest influence on melanoma‐specific survival (MSS) were positive SLN status and mitotic rate. In conclusion, SLN biopsy should probably be offered if the primary tumor has LVI. MS is an adverse prognostic factor for RFS, but its influence on outcome is modest. Ulceration width predicts RFS but loses its independent prognostic significance for MSS when adjusting for currently used clinicopathological factors. In view of its impact on MSS, mitotic rate should be recorded for cutaneous invasive melanomas across all T categories.

## Introduction

The accurate assessment and documentation of relevant clinicopathological features are essential for the optimal management of patients with cutaneous melanoma. Several studies have shown that the presence or absence of ulceration predicts recurrence and survival 1. In analyses leading to the 7th and 8th editions of the American Joint Committee on Cancer (AJCC) melanoma staging system, the presence of ulceration was shown to be an independent adverse predictor of survival in stages I–III melanoma and thus was incorporated into the AJCC staging system 2. In 1980, Balch et al. were the first to report that ulceration width, which is the diameter of ulceration in primary tumor, was significantly correlated with survival 3. Several subsequent studies confirmed that the extent of ulceration measured either as a diameter of ulceration ranging from 2.0 mm to 6.0 mm 3, 4, 5, 6, 7, 8 or a percentage of ulceration relative to the underlying dermal‐invasive component 8, 9, 10 predicted recurrence and survival. However, since most of these reports were published prior to the sentinel lymph node (SLN) era, it is clinically relevant to assess the impact of ulceration width along with other potential prognostic factors in the contemporary era. Associations between the presence of lymphovascular invasion (LVI) and SLN positivity/regional nodal involvement were significant in many 11, 12, 13, 14, 15, 16, but not all 17 studies by univariate analysis, with less clear results in multivariable analyses 13, 14, 15. Association between LVI and recurrence or survival has been consistently significant in univariate analyses 11, 12, 18, 19, but the results of multivariable analyses have been conflicting. Meanwhile, associations between the presence of microscopic satellitosis (MS) and disease‐free survival (DFS)/recurrence‐free survival (RFS) have been consistently significant in several studies. However, whether the presence of MS predicts SLN positivity or melanoma‐specific survival (MSS) is less clear. Perineural invasion (PNI) may be associated with an increased risk of local recurrence 20. However, its impact on SLN positivity or MSS is unclear. Mitotic rate was used for T1 subcategorization in the 7th edition AJCC melanoma staging system, but it was excluded from the latest 8th edition 21. Therefore, the purpose of this study was to evaluate the importance of ulceration width and other important pathological measurements such as LVI, MS, PNI, and mitotic rate in terms of SLN positivity, RFS, and MSS for patients with cutaneous melanoma in the SLN era.

## Methods

### Patients

We performed a single‐institutional retrospective study at The University of Texas MD Anderson Cancer Center (MD Anderson) using the prospectively collected pathology and clinical database. In our institution, in general, all the patients with invasive melanoma with AJCC 7th edition stages T1b and above, as well as at least T1a (i.e., tumor with Breslow thickness <1 mm without mitotic rate or ulceration, but was transected at the base), are offered SLN biopsy in addition to wide local excision of the lesion. For this study, we identified all patients with primary invasive cutaneous melanoma who underwent SLN biopsy in the period between 1 January 2003, and 31 December 2008, at our institute. Wide local excision of the primary melanoma was performed with margins appropriate for the tumor thickness. Lymphatic mapping and SLN biopsy were performed as described previously 22. Patients with a positive SLN were offered completion lymph node dissection (CLND) during a discussion of their treatment plan. Permission to perform this study and a waiver for informed consent were obtained from the MD Anderson Institutional Review Board.

### Clinicopathological factors

**Figure 1:**
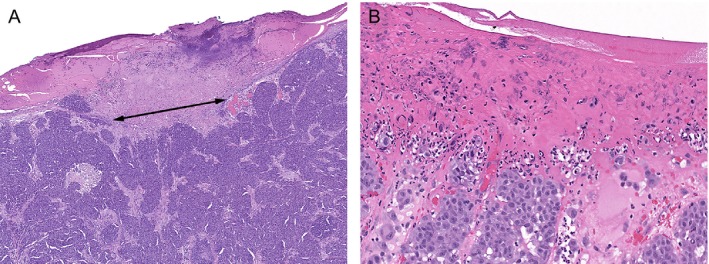
Measurement of width of ulceration (mm) in cutaneous melanoma (A). Ulceration showing the absence of intact epidermis overlying primary invasive melanoma, associated with serum crust and fibrinous exudate (B).

The pathological parameters used in this study were consistent with the required data elements for the reporting of cutaneous invasive melanoma using the College of American Pathologists (CAP) protocol 23. We additionally included ulceration width and stratified mitotic rate into four groups (<1/mm^2^ (equal to zero), 1/mm^2^, 2–5/mm^2^, and >5/mm^2^) based on the distribution of the patients in our cohort. Ulceration was defined as a loss of all layers of the epidermis overlying the invasive component of the primary melanoma with associated scale crust and fibrin deposit 24. We measured ulceration width linearly in millimeters from one edge to the other edge using an ocular micrometer (Fig. 1). LVI was defined as vascular invasion with melanoma cells within lymphatics or blood vessels or both in intratumoral or peri‐tumoral area 23 In some cases, immunohistochemistry was used to highlight the presence of endothelial cells around the tumor cells 12. MS was defined as the presence of microscopic cutaneous and/or subcutaneous metastasis adjacent or deep to a primary melanoma separated from primary tumor by normal dermis or subcutis. 21. PNI was defined as the presence of melanoma cells located adjacent to nerve sheath, usually circumferentially 21. Mitotic rate was defined as the number of mitotic figures per square millimeter in the dermal component. Pathologic evaluation of the SLN was performed as described previously 25.

### Statistical methods

The associations between the clinicopathological factors and SLN positivity or RFS/MSS were assessed using univariate and multivariable logistic regression models or Cox proportional hazard regression models, respectively. The final multivariable model was determined using stepwise selection with an entrance criterion of *P* < 0.10 and an exit criterion of *P* ≥ 0.05. Only patients with complete information for each analysis were included in each model. To determine the optimal cutoff value for ulceration width, every possible cutoff point was evaluated using patient outcome. Patients were divided into two groups based on each cutoff value. The cutoff point that yielded the most significant difference between the ulcerated groups was chosen as the optimal value 26. RFS was computed from the date of the SLN biopsy to the date of disease recurrence or death. Patients who were alive and had not recurred by the last follow‐up date were censored. MSS was computed from the date of the SLN biopsy to the date of death due to melanoma. Patients who died from other causes as well as those who were alive at the last follow‐up were censored. The Kaplan–Meier method was used to estimate RFS and MSS and differences between groups were assessed using the log‐rank test. All statistical analyses were performed using SAS 9.3 for Windows (Copyright © 2011 by SAS Institute Inc., Cary, NC). A significance level of 5% was used in all the statistical tests and no adjustments were made for multiple testing.

## Results

We identified 1898 eligible patients. In 8 of the 1898 patients, SLN biopsy was performed twice within the study period, and only the pathological data of more advanced stage tumor in each patient were included. The median tumor thickness was 1.25 mm (range, 0.12–51.0 mm). Ulceration was present in primary tumors of 371 patients (20%), and among the ulcerated primary tumors, the median ulceration width was 3.0 mm (range, 0.05–50.0 mm). The optimal cutoff point of the ulceration width with respect to RFS and MSS was determined to be 7.2 and 7.45 mm, respectively. Based on ease of potential clinical use, we rounded these cutoff points to 7.0 mm. LVI, MS, and PNI were observed in 82 patients (4%), 52 patients (3%), and 61 patients (3%), respectively. Mitotic rate (<1/mm^2^, 1/mm^2^, 2–5/mm^2^, >5/mm^2^) was observed in 575 (30%), 357 (19%), 566 (30%), and 305 (16%) patients, respectively.

The SLN was histologically positive in 336 patients (18%), and CLND was performed in 308 (92%) of these 336 patients. Systemic adjuvant therapy was administrated in 164 (9%) of 1898 overall patients, and 136 (40%) of 336 patients with positive SLNs. The agent most frequently used was interferon alfa, which was used in 73% of the patients who received adjuvant systemic therapy. The median follow‐up was 6.7 years (range, 0–11.4 years) and 7.3 years (range, 0–11.2 years) for patients who were alive at last follow‐up. Four hundred eight‐six of 1883 (26%) patients experienced recurrence and/or death (RFS) while 12% (221 of 1878) patients experienced events of melanoma deaths (MSS).

### Factors associated with SLN positivity

In multivariable analysis, the dominant independent predictors of a positive SLN were a thicker tumor thickness and the presence of LVI. In contrast, patients with an upper extremity primary tumor site, or lentigo maligna melanoma (LMM) histological subtype and elderly patients independently, had a lower SLN positivity rate (Table 1).

**Table 1:** Factors associated with SLN positivity

	Univariate			Multivariable	
	*n*	OR (95% CI)	*P*‐value	OR (95% CI)	*P*‐value
Gender					
Female (Ref)	789	–	–		
Male	1109	1.29 (1.01, 1.64)	0.042	NI	
Age (years)					
<40 (Ref)	385	–	–	–	–
40–60	829	1.10 (0.80, 1.51)	0.57	1.01 (0.70, 1.45)	0.96
>60	684	1.05 (0.75, 1.46)	0.78	0.69 (0.47, 1.01)	0.058
Primary site					
Trunk (Ref)	794	–	–	–	–
Head and neck	350	1.08 (0.78, 1.50)	0.63	0.77 (0.53, 1.12)	0.17
Upper limb	362	0.62 (0.43, 0.90)	0.013	0.60 (0.40, 0.89)	0.011
Lower limb	392	1.44 (1.07, 1.94)	0.017	1.34 (0.96, 1.89)	0.09
Subtype					
SSM (Ref)	1135	–	–	NI	
NM	319	2.45 (1.82, 3.30)	<0.001		
LMM	118	0.40 (0.18, 0.87)	0.021		
ALM	89	3.55 (2.23, 5.65)	<0.001		
Unclassified	221	1.85 (1.29, 2.64)	<0.001		
Tumor thickness					
T1a (Ref)	428	–	–	–	–
T1b	286	4.46 (2.05, 9.70)	<0.001	4.53 (2.08, 9.87)	<0.001
T2	552	10.05 (5.01, 20.15)	<0.001	10.49 (5.20, 21.15)	<0.001
T3	391	22.14 (11.06, 44.29)	<0.001	22.84 (11.29, 46.21)	<0.001
T4	189	32.01 (15.56, 65.84)	<0.001	33.18 (15.86, 69.40)	<0.001
Ulceration width (mm)					
0 (absent) (Ref)	1523	–	–		
>0–7.0	302	2.05 (1.53, 2.74)	<0.001	NI	
>7.0	63	3.99 (2.37, 6.72)	<0.001		
Mitotic rate					
<1/mm^2^ (Ref)	575	–	–	NI	
1/mm^2^	357	2.89 (1.82, 4.59)	<0.001		
2–5/mm^2^	566	5.32 (3.54, 7.97)	<0.001		
>5/mm^2^	305	8.65 (5.64, 13.28)	<0.001		
Lymphovascular invasion					
Absent (Ref)	1746	–	–	–	–
Present	82	5.47 (3.49, 8.60)	<0.001	2.87 (1.76, 4.70)	<0.001
Microscopic satellitosis					
Absent (Ref)	1772	–	–		
Present	52	3.31 (1.88, 5.84)	<0.001	NI	
Perineural invasion					
Absent (Ref)	1762	–	–		
Present	61	1.68 (0.94, 3.01)	0.08	NI	

ALM, acral lentiginous melanoma; CI, confidence interval; LMM, lentigo maligna melanoma; NI, not included in the final model (*P* ≥ 0.05); NM, nodular melanoma; OR, odds ratio; Ref, reference group; SSM, superficial spreading melanoma; The numbers in some groups do not total 1898 because data were not available for some patients.

### Factors associated with RFS

**Figure 2:**
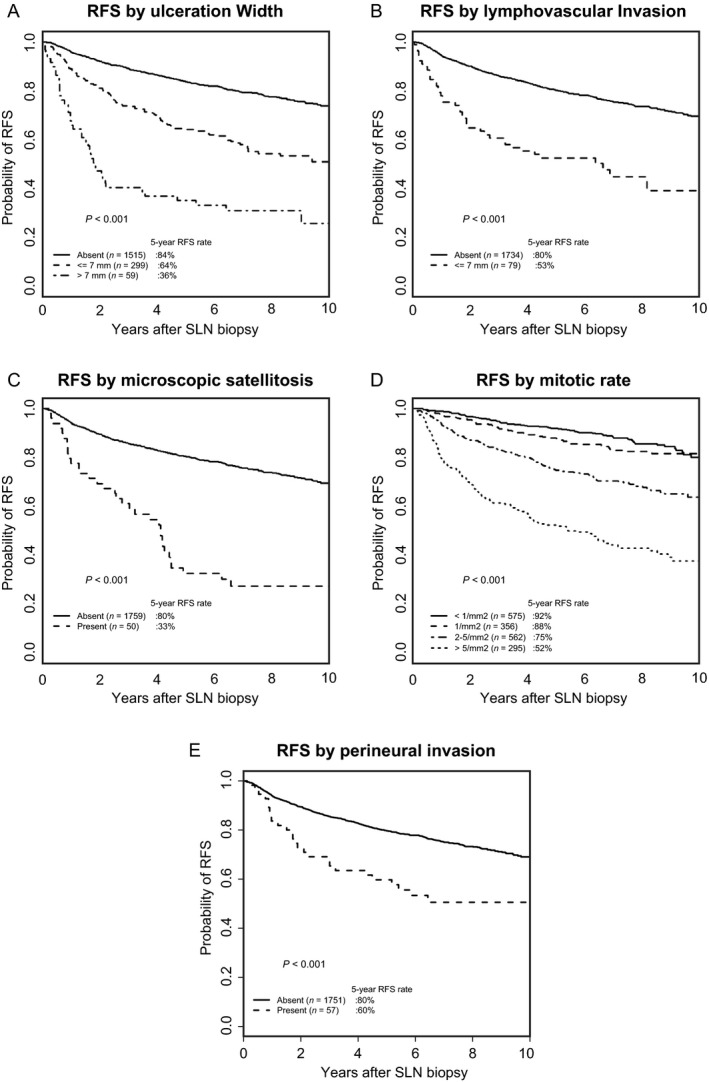
Recurrence‐free survival according to ulceration width (A), lymphovascular invasion (B), microscopic satellitosis (C), mitotic rate (D), and perineural invasion (E).

In multivariable analysis, the dominant independent predictors of a higher risk of recurrence were a thicker tumor thickness, older age, and the positive SLN status; patients who had a higher mitotic rate, head and neck primary tumor site, ALM subtype, presence of MS, larger ulceration width, and patients who were male were also significantly associated with a shorter RFS. Conversely, patients with an upper extremity primary tumor site had a longer RFS. PNI was significantly associated with RFS only in univariate analysis (Table 2). Survival curves for RFS according to ulceration width, LVI, MS, PNI, and mitotic rate are shown in Figure 2.

**Table 2:** Factors associated with recurrence‐free and melanoma‐specific survival

	RFS					MSS				
	Univariate			Multivariable		Univariate			Multivariable	
	*n*	HR (95% CI)	*P*‐value	HR (95% CI)	*P*‐value	*n*	HR (95% CI)	*P*‐value	HR (95% CI)	*P*‐value
Gender										
Female (Ref)	785	–	–	–	–	781	–	–		
Male	1098	1.82 (1.50, 2.21)	<0.001	1.30 (1.05, 1.61)	0.017	1097	1.65 (1.24, 2.19)	<0.001	NI	
Age (years)										
<40 (Ref)	382	–	–	–	–	382	–	–	–	–
40–60	826	1.97 (1.38, 2.82)	<0.001	1.67 (1.14, 2.45)	0.009	824	2.46 (1.47, 4.14)	<0.001	1.88 (1.09, 3.24)	0.022
>60	675	5.39 (3.82, 7.58)	<0.001	4.03 (2.77, 5.85)	<0.001	672	4.39 (2.63, 7.31)	<0.001	3.23 (1.88, 5.55)	<0.001
Primary site										
Trunk (Ref)	790	–	–	–	–	790	–	–	–	–
Head Neck	344	2.02 (1.62, 2.52)	<0.001	1.34 (1.04, 1.72)	0.024	344	1.82 (1.31, 2.53)	<0.001	1.17 (0.80, 1.72)	0.42
Upper limb	359	0.84 (0.64, 1.10)	0.21	0.74 (0.55, 0.99)	0.040	356	0.56 (0.35, 0.89)	0.015	0.50 (0.31, 0.83)	0.007
Lower limb	390	1.06 (0.82, 1.35)	0.67	0.79 (0.58, 1.07)	0.13	388	1.22 (0.86, 1.72)	0.26	0.66 (0.42, 1.01)	0.06
Subtype										
SSM (Ref)	1127	–	–	–	–	1127	–	–	–	–
NM	316	1.80 (1.43, 2.28)	<0.001	0.77 (0.59, 1.01)	0.06	312	1.97 (1.39, 2.79)	<0.001	0.72 (0.49, 1.06)	0.10
LMM	117	2.11 (1.50, 2.96)	<0.001	1.32 (0.90, 1.94)	0.15	116	1.69 (0.95, 3.02)	0.08	1.31 (0.69, 2.47)	0.41
ALM	89	3.38 (2.45, 4.67)	<0.001	2.09 (1.40, 3.13)	<0.001	88	5.04 (3.32, 7.65)	<0.001	3.19 (1.87, 5.45)	<0.001
Unclassified	218	1.84 (1.41, 2.40)	<0.001	1.02 (0.76, 1.38)	0.90	219	2.10 (1.42, 3.09)	<0.001	0.95 (0.61, 1.48)	0.81
Tumor thickness										
T1a (Ref)	428	–	–	–	–	428	–	–	–	–
T1b	286	1.24 (0.80, 1.92)	0.34	0.84 (0.47, 1.50)	0.55	285	3.58 (1.12, 11.41)	0.031	1.79 (0.47, 6.87)	0.39
T2	550	2.51 (1.78, 3.54)	<0.001	1.20 (0.73, 1.96)	0.47	549	11.18 (4.06, 30.80)	<0.001	3.69 (1.12, 12.20)	0.032
T3	387	5.78 (4.14, 8.07)	<0.001	1.82 (1.08, 3.07)	0.025	386	28.67 (10.53, 78.04)	<0.001	5.54 (1.64, 18.66)	0.006
T4	180	7.57 (5.28, 10.87)	<0.001	2.11 (1.20, 3.70)	0.010	178	46.26 (16.80, 127.37)	<0.001	8.31 (2.40, 28.78)	<0.001
Ulceration width (mm)										
0 (absent) (Ref)	1515	–	–	–	–	1510	–	–		
≤7.0	299	2.36 (1.92, 2.91)	<0.001	1.14 (0.89, 1.45)	0.29	298	2.64 (1.94, 3.59)	<0.001	NI	
>7.0	59	5.80 (4.19, 8.04)	<0.001	1.76 (1.19, 2.62)	0.005	60	7.84 (5.14, 11.96)	<0.001		
Mitotic rate										
<1/mm^2^ (Ref)	575	–	–	–	–	575	–	–	–	–
1/mm^2^	356	1.26 (0.89, 1.79)	0.19	1.10 (0.69, 1.76)	0.68	355	2.19 (1.10, 4.38)	0.026	1.29 (0.58, 2.88)	0.54
2–5/mm^2^	562	2.63 (2.00, 3.46)	<0.001	1.72 (1.14, 2.58)	0.009	557	6.53 (3.71, 11.50)	<0.001	2.68 (1.33, 5.37)	0.006
>5/mm^2^	295	6.14 (4.65, 8.10)	<0.001	2.46 (1.60, 3.78)	<0.001	296	15.97 (9.09, 28.05)	<0.001	4.11 (2.02, 8.35)	<0.001
Lymphovascular invasion										
Absent (Ref)	1734	–	–			1729	–	–		
Present	79	2.90 (2.10, 4.01)	<0.001	NI		79	4.00 (2.67, 6.00)	<0.001	NI	
Microscopic satellitosis										
Absent (Ref)	1759	–	–	–	–	1754	–	–		
Present	50	4.09 (2.88, 5.80)	<0.001	1.63 (1.11, 2.37)	0.012	50	4.08 (2.51, 6.62)	<0.001	NI	
Perineural invasion										
Absent (Ref)	1751	–	–			1747	–	–		
Present	57	2.32 (1.56, 3.45)	<0.001	NI		56	3.13 (1.85, 5.29)	<0.001	NI	
Number of positive SLNs										
0 (Negative) (Ref)	1555	–	–	–	–	1550	–	–	–	–
1	238	2.82 (2.28, 3.49)	<0.001	2.06 (1.63, 2.62)	<0.001	236	5.00 (3.72, 6.72)	<0.001	2.79 (2.00, 3.88)	<0.001
>1	90	3.81 (2.81, 5.16)	<0.001	2.34 (1.67, 3.27)	<0.001	92	8.15 (5.63, 11.80)	<0.001	4.20 (2.78, 6.32)	<0.001
Adjuvant systemic therapy										
Absent (Ref)	1717	–	–			1710	–	–		
Present	162	1.63 (1.25, 2.13)	<0.001	NI		162	2.75 (1.98, 3.81)	<0.001	NI	

ALM, acral lentiginous melanoma; CI, confidence interval; HR, hazard ratio; LMM, lentigo maligna melanoma; MSS, melanoma‐specific survival; NI, not included in the final model (*P* ≥ 0.05); NM, nodular melanoma; Ref, reference group; RFS, recurrence‐free survival; SSM, superficial spreading melanoma; The numbers in some groups do not total 1898 because data were not available for some patients.

### Factors associated with MSS

**Figure 3:**
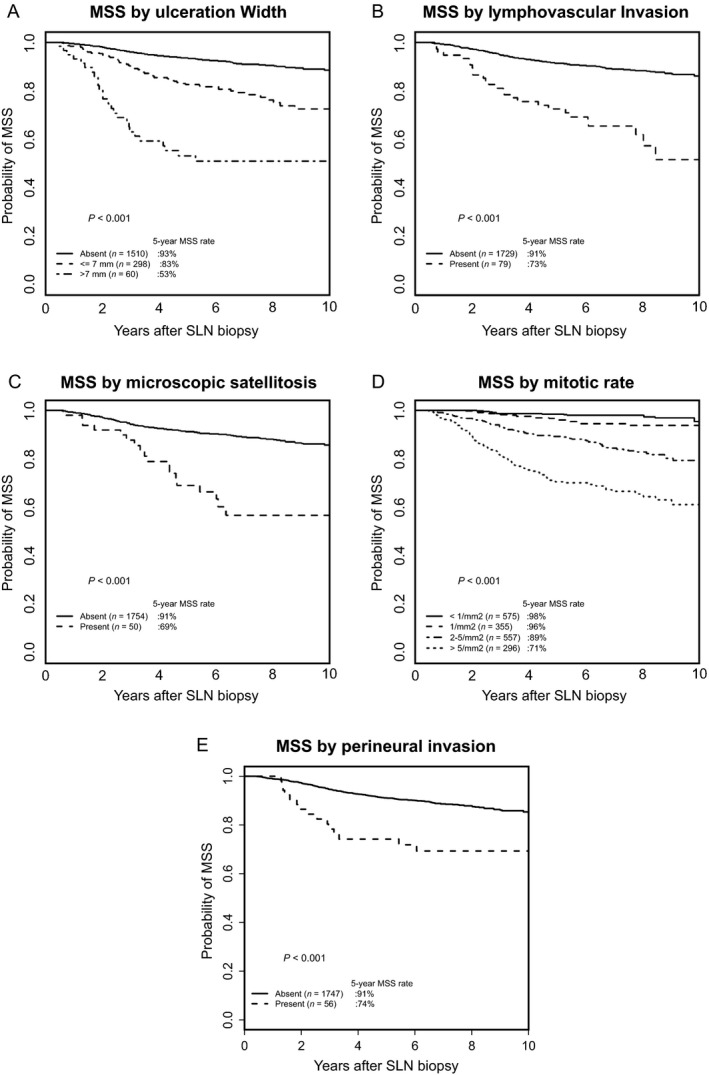
Melanoma‐specific survival according to ulceration width (A), lymphovascular invasion (B), microscopic satellitosis (C), mitotic rate (D), and perineural invasion (E).

In multivariable analysis, the strongest independent predictors for worsening of MSS were the positive SLN status and a higher mitotic rate; patients who had thicker tumor thickness, ALM histological subtype, and older age were also associated with worse MSS. In contrast, patients with upper extremity primary tumor site had a lower risk of melanoma‐specific death. PNI was significantly associated with MSS only in univariate analysis (Table 2). Survival curves for MSS according to ulceration width, LVI, MS, PNI, and mitotic rate are shown in Figure 3.

## Discussion

In this study, a larger ulceration width (>7.0 mm) predicted RFS, but did not independently predict SLN positivity or MSS when adjusting for other factors. Instead of the actual measurement of the ulceration width in millimeters, the concept of the percentage ulceration has also been investigated in three studies 8, 9, 10. The percentage ulceration was defined as the percentage of ulceration relative to the surface diameter of the vertical growth phase of the tumor 9 or the ratio of the greatest diameter of the ulceration to that of the dermal‐invasive component 8, 10. However, we did not incorporate percentage ulceration as it is not always easy to determine the edge of the dermal‐invasive component due to the presence of associated melanocytic nevus or the fact that the entire dermal‐invasive component is not always included in the initial biopsy specimen. In previous reports, association between ulceration width and DFS/RFS was consistently significant in both univariate and multivariable analyses 5, 8. On the other hand, although the association between ulceration width and OS/MSS was consistently significant in univariate analyses 3, 6, 7, 8, the results of multivariable analyses varied, with no significance observed in some studies 6, 7 and significance observed in another 8. Hout et al. studied clinicopathological and follow‐up data on 4661 patients and showed that larger ulceration width (>5 mm) or higher percentage ulceration (>70%) is associated with a significantly higher risk of death 8. Although the mechanism for ulceration overlying a primary melanoma is not yet well understood, rapid tumor growth and interruption of the dermal vascular supply are thought to cause necrosis of the epidermis 3. Mitotic rate reflects tumor proliferation; Hout et al. showed a significant association between mitotic rate and ulceration width. Considering the correlation between a larger ulceration width and a higher mitotic rate, whether ulceration width becomes significant in the multivariable analyses can be influenced by how the mitotic rate was handled. Hout et al. used dichotomous categories (<1/mm^2^, ≥1/mm^2^) and demonstrated significance in a multivariable analysis, whereas the present study used multiple categories (<1/mm^2^, 1/mm^2^, 2–5/mm^2^, and >5/mm^2^) and did not show a significant correlation of ulceration width. These results suggest that larger ulceration width, which is associated with a higher mitotic rate, may lose its independent prognostic significance for survival when mitotic rate is included across its continuum using multiple categories. Another important finding in this study was that mitotic rate was the second most powerful predictor of MSS after the positive SLN status. The importance of mitotic rate was also confirmed in both localized cutaneous melanoma 27 and cutaneous melanoma with nodal micrometastases 28. Although mitotic rate was excluded from the 8th edition AJCC melanoma staging system 21, considering its strong impact on MSS, it will likely be incorporated again into future staging systems and prognostic models and tools; mitotic rate should be collected for all patients with primary cutaneous invasive melanoma across all T categories.

The presence of LVI was the second strongest independent predictor for SLN positivity after thicker tumor thickness, but it did not independently predict RFS or MSS when adjusting for other factors. LVI is defined as the presence of melanoma cells within lymphatics or blood vessels, and Doeden et al. showed that the former type of vascular invasion occurred more frequently (lymphatics 16% vs. blood vessels 3%, *P* = 0.001) 14. LVI is infrequently seen using routine H&E staining, ranging from 3 to 16% of samples 11, 15, 17, 18, 19. The sensitivity of detection of lymphatic invasion can be increased using immunohistochemistry, ranging from 16 to 33% with D2‐40 (podoplanin) 12, 13, 14, 16, and ranging from 37 to 43% with dual staining for D2‐40 and S‐100 17, or D2‐40 and MITF 16, which makes it easier to distinguish melanoma cells from hematopoietic cells within lymphatic vessels. Although the results of multivariable analyses varied in previously reported studies 13, 14, 15, the present study with the largest sample size confirmed a significant association between LVI and SLN positivity. Associations between LVI and recurrence or survival have been consistently significant in univariate analyses 11, 12, 18, 19, but the results of multivariable analyses have been conflicting. Unlike the present study, SLN status was not included as a covariate in multivariable analyses in several studies, even though patients with nodal involvement were included 11, 12, 18 or patients that predated the use of SLN biopsy did not undergo prophylactic regional node dissection 19. Considering the strong correlation between the presence of LVI and SLN positivity, it is not surprising that the results of the multivariable analyses depended on whether SLN status was included in the model or whether the population included patients with nodal involvement.

The presence of MS predicted RFS, but did not independently predict SLN positivity or MSS when adjusting for other pathological measures. In previous studies reported by Rao et al. 29, Kimsey et al. 30, and authors of references therein, the incidence of MS ranged from 3 to 19%. The incidence of regional nodal involvement was higher if MS was present, ranging from 40 to 68%, compared with that if MS was absent, ranging from 7 to 23%. However, the present study did not show an association between MS and SLN positivity in multivariable analyses. Association between the presence of MS and DFS/RFS has been consistently significant in univariate 18, 29, 30 and multivariable analyses, with HRs ranging from 1.58 to 2.82 18, 29. MS, which is a component of stage III in the 8th edition AJCC melanoma staging system, is confirmed as an adverse prognostic factor for increased risk of recurrence. Association between the presence of MS and overall survival has been generally consistently significant in univariate analyses 18, 29, 30; however, the results of the multivariable analyses have varied, with HRs ranging from 1.80 to 1.98 18, 29. The present study similarly did not demonstrate its independent prognostic significance for MSS.

PNI is commonly seen in desmoplastic melanoma but it is also seen in other types of melanoma. On the other hand, melanoma with desmoplastic feature does not always contain PNI and it is often combined with other features such as LMM subtype. The presence of PNI is associated with increased risk of local recurrence 20. In the present study, the presence of PNI was significantly associated with both RFS and MSS by univariate analyses, but not by multivariable analysis. Surgery followed by postoperative radiotherapy appears to provide superior local control compared with surgery alone for patients with desmoplastic melanoma 31, and radiotherapy for desmoplastic melanoma was independently associated with improved local control 32. In our cohort, 22 (36%) of 61 patients who had melanoma with PNI received postoperative radiotherapy for the primary site.

Older age was also associated with worse RFS and MSS whereas it was not associated with SLN positivity. In a recently reported multi‐institutional study of 1524 patients with cutaneous melanoma greater than 1 mm in thickness also showed a similar result; older age was not associated with SLN positivity but was associated with worse DFS and OS 33.

In conclusion, SLN biopsy should likely be offered if the primary tumor has LVI, as LVI was the second strongest predictor for SLN positivity after tumor thickness. MS is an adverse prognostic factor for RFS, but its influence on outcome is modest. Ulceration width predicts RFS but may lose its independent prognostic significance for MSS when adjusting for other contemporary clinicopathologic factors. As mitotic rate was the second strongest predictor for MSS after the positive SLN status, we recommend continuing reporting the mitotic rate for primary cutaneous invasive melanomas across all T categories.

*Limitation of the study*: relatively short median follow‐up period.

## Conflict of Interest

The authors have no conflicts of interests to declare.
